# Amyotrophic Lateral Sclerosis: The State of the Art on Treatments and the Therapeutic Role of the Intestinal Microbiome in Human Studies

**DOI:** 10.3390/ijms27041655

**Published:** 2026-02-08

**Authors:** Ondřej Ptáček, Zdeněk Musil, Giulia Guarnieri, Alena Vrbacká, Pavla Moudrá, Aneta Zlámalová, Petra Röszlerová, Michal Tonhajzer, Vladimír Musil, Annamaria Morelli, Petr Zach

**Affiliations:** 1 Institute of Biology and Medical Genetics, First Faculty of Medicine, Charles University and General University Hospital, 128 21 Prague, Czech Republic; ondrej.a.ptacek@gmail.com; 2 Department of Anatomy, Third Faculty of Medicine, Charles University, 100 00 Prague, Czech Republic; 3 Department of Experimental and Clinical Medicine, University of Florence, 50134 Florence, Italy; giulia.guarnieri@unifi.it (G.G.); a.morelli@unifi.it (A.M.); 4 Department of Paediatrics and Inherited Metabolic Disorders, First Faculty of Medicine, Charles University and General University Hospital, 128 21 Prague, Czech Republic; acizk@lf1.cuni.cz; 5 Institute of Laboratory Diagnostics and Public Health, Faculty of Health and Social Sciences, University of South Bohemia, 370 11 České Budějovice, Czech Republic; pmoudra@zsf.jcu.cz (P.M.); zlamalova@zsf.jcu.cz (A.Z.); javurkova@zsf.jcu.cz (P.R.); 6 Faculty of Mechanical Engineering, Czech Technical University, 160 00 Prague, Czech Republic; tonhamic@cvut.cz; 7 Centre of Scientific Information, Third Faculty of Medicine, Charles University, 100 00 Prague, Czech Republic; vladimir.musil@lf3.cuni.cz; 8 Department of Anatomy, Second Faculty of Medicine, Charles University, 150 06 Prague, Czech Republic; 9 Institute of Physiotherapy and Selected Medical Disciplines, Faculty of Health and Social Sciences, University of South Bohemia, 370 11 České Budějovice, Czech Republic

**Keywords:** amyotrophic lateral sclerosis, ALS, faecal microbiota transplantation, FMT, intestine, gut microbiome, human

## Abstract

Amyotrophic lateral sclerosis (ALS) is a common neurodegenerative disorder; to date, there is no long-term effective treatment. Recently, a relationship has been discovered between the human intestinal microbiome and the pathogenesis of ALS, on which basis faecal microbiota transplantation (FMT) has been proposed as a potential treatment for ALS. In this review, we compare three existing case studies examining the effect of FMT on the course of ALS, highlighting differences in methodology and results. In two of the studies, a halt in the progression of ALS symptoms was observed following FMT, accompanied by improvement in patient health. However, in the third and largest study, no significant effect of FMT was observed. The possible explanation for this discrepancy may be the intentional depletion of intestinal microorganisms using antibiotics prior to FMT in the third study. Future studies and/or completion of the ongoing clinical studies will help clarify the therapeutic effectiveness of FMT in ALS patients.

## 1. Introduction

Amyotrophic lateral sclerosis (ALS) is a multifactorial progressive neurodegenerative disease causing degeneration and eventual destruction of both upper and lower motor neurons, which then leads to gradual loss of skeletal muscle strength, paralysis and respiratory failure in the terminal stages of the disease [1]. This review summarises the findings of studies with published results focusing on the effect of faecal microbiota transplantation (FMT) in human patients with ALS.

### 1.1. ALS Features, Diagnosis and Current Treatments

ALS is mostly diagnosed between 40 and 70 years of age, depending on the specific type of the disease [2]. The disease often begins with a focal onset, from which ALS subsequently spreads into other body regions. Due to the progression of ALS, respiratory muscles are atrophied, which limits the lifespan of patients to 2–5 years after the initial diagnosis. Although the progression of ALS is highly variable, a lifespan of more than 10 years after the diagnosis was recorded only in about 10% of patients [3]. Higher age of diagnosis and the bulbar form of ALS were associated with a worse prognosis, whereas in younger patients and in some spinal forms of the disease, the progression of ALS was slower [4].

Although ALS primarily affects the motoric system, about half of the patients also exhibit extramotoric symptoms, for example, cognitive dysfunction, behavioural changes, emotional liability, pseudobulbar effects, anxious and depressive symptoms, sleep disorders, fatigue, and pain [3]. Another frequent problem is sialorrhea (excessive salivation) and constipation, which are related to the limited mobility and to possible changes to intestinal motility caused by ALS. These nonmotoric symptoms negatively affect the quality of life of patients and might indirectly influence the prognosis of the disease [5]. The complex clinical presentation of ALS not only consists of the progressive motor paralysis, but also of the above-mentioned systemic symptoms, which underlines the necessity of multidisciplinary medical care focused on nutrition, respiration, communication, psychology, and palliative support, among others [6].

### 1.2. Health Assessment of ALS Patients

In order to assess the severity and progress of ALS in a clinical environment, several methods are utilised. The most frequently used is Amyotrophic Lateral Sclerosis Functional Rating Scale-Revised (ALSFRS-R), which assesses functions such as speech, salivation, swallowing, handwriting, self-feeding, dressing and personal hygiene, turning in bed, climbing stairs, dyspnoea, orthopnoea, and respiratory insufficiency. Each is graded from 0 to 4, with a higher total score indicating better patient health and prognosis [7]. An alternative method for ALS evaluation is the ALSAQ-40, the 40-item Amyotrophic Lateral Sclerosis Assessment Questionnaire [8], a questionnaire consisting of 40 questions focused on the patient’s physical mobility, eating, daily activities, independence, communication, and emotional responsiveness, measured on a scale from 0 to 100, with a maximum achievable total of 500 points, where higher scores correspond to lower quality of life.

### 1.3. Aetiology of ALS

The global incidence of ALS is approximately 1–2.6 cases per 100,000 people annually, while the prevalence is approximately 6 cases per 100,000 inhabitants [9], although accurate assessment of the global burden of ALS is difficult due to limited data from certain regions of the world, such as Africa and Oceania [10]. Specifically in Europe, the incidence of 2.31 per 100,000 and the prevalence of 6.22 per 100,000 was recorded [11].

The clinical prognosis of ALS is heavily influenced by the patient’s genotype and by the specific genetic mutations involved in the pathogenesis of the disease. Approximately 5–10% of cases are attributed to the familial, inherited form of ALS, while the remaining 90–95% of cases are denoted as sporadic ALS [12], with mutations identified in genes *SOD1*, *TARDBP*, *FUS* and *C9orf72* being the most extensively studied in both variants of the disease.

Although genetic factors play a significant role in ALS, in recent years, the research has focused on epigenetic and environmental factors contributing to ALS [13,14]. Besides other nongenetic pathogenic factors of ALS, dysbiosis of the intestinal microbiome, which by itself might be induced by environmental factors (use of antibiotics, physical stress, viral infection, and exposure to toxins), was found to significantly influence the progress of the disease [15]. Dysbiosis of the microbiome was also connected to elevated levels of proinflammatory cytokines and the activation of microglia in both the spinal cord and brain, which would indicate the existence of a potential neuroimmune microbiome–gut–brain pathway contributing to the progression of ALS [16].

### 1.4. Role of Human Intestinal Microbiome in ALS

The intestinal microbiome is a complete set of genomes of bacteria, archaea, and viruses native to the gastrointestinal tract of a host organism, whereas the complex community itself is collectively referred to as microbiota [17]. Intestinal bacteria have a key role in the defence against pathogens and the maintenance of immune and metabolic homeostasis [18]. Human intestinal microbiome exhibits a high heterogeneity, while only about 1/3 of microbial species are shared by the global human population [19]. The heterogeneity of the human intestinal microbiome is a product of several environmental factors, such as past viral and bacterial infections, treatment via antibiotics, nutrition, ingestion of probiotics and prebiotics, environmental stressors, age of the individuals, and their genetic makeup [20].

Intestinal microbiotas are an important part of the host organism due to their multifaceted involvement with other organs via nervous, endocrine, humoral, immunological, and metabolic pathways. Any alteration to the microbial community could not only cause problems limited to the intestines, but also health issues concerning other organs, although the exact mechanism of the closer interaction of microbes, intestines, and other organs is not completely understood to date [21]. In regard to the composition of the human intestinal microbiome, bacterial genera *Firmicutes*, *Bacteroides*, *Actinobacteria*, *Proteobacteria* and *Verrucomicrobia* compose a significant part of the intestinal bacterial population [22]. The first step in the identification of interactions between microbes and their hosts is a description of an ideal, balanced composition of the intestinal microbiome and the role of its variations in the pathogenesis of diseases [23]. In a recent pilot study [24], the composition of the intestinal microbiome was examined in patients suffering from ALS in the early stage of the disease. The study comprised 28 participants, of whom 16 were diagnosed with ALS, and 12 served as healthy controls, from whom samples of stool were isolated and subsequently analysed via 16S rRNA genetic sequencing. No significant difference was found in the complete biodiversity of the intestinal microbiome between patients and the control group, although a variation was detected in the quantitative representation of each individual bacterial species population. In patients with ALS, a higher presence of bacterial phyla *Fusobacteria* and *Acidobacteria* was detected, and in terms of genera higher presence of *Enterobacter*, *Clostridium*, *Veillonella*, *Dialister*, *Turicibacter* and *Acidaminococcus* was also observed. On the other hand, the presence of the genera *Prevotella*, *Lactobacillus* and *Butyricimonas* was diminished in comparison with the control group [24].

### 1.5. Routine Treatments of ALS

Contemporarily, there are several treatments of ALS in use in order to manage its symptoms. Unfortunately, due to the above-mentioned heterogeneity of ALS, its treatment must be approached mostly on an individual basis. Specifically, the variance in onset of symptoms and the degree of their severity must be considered when prescribing the treatment, and even then, the treatments might be met with varying effects on at first glance highly similar cases.

### 1.6. Sialorrhea

Sialorrhea, ptyalism, or an excessive apparent production of saliva, is a frequent health complication found in patients suffering from ALS. It is not caused by an increased stimulation of salivary glands resulting in an increased production of saliva and mucus [25], but by the patient’s body losing the ability to manage its usual secretion instead. As the main mechanism by which the body manages the excessive saliva, swallowing is affected by progressive oesophageal paralysis, the main presentation of ALS.

In order to manage the excessive salivation, several therapies are either already in use or are experimented with [26]. Administration of anticholinergic drugs, which work on the basis of neurotransmitter blockers affecting the sympathetic and parasympathetic nervous system, is one of such treatments [27]. For example, hyoscine hydrobromide is an anticholinergic drug commonly used in ALS patients. Similarly, a neurectomy of the tympanic plexus nerve prohibits the transmission of nervous signals responsible for the production of saliva, which in effect decreases salivation.

Another possible treatment of sialorrhea can be aimed directly at the salivary glands. An experimental study in which parotid and submandibular glands were injected with botulinum toxins A and B found no significant effect on sialorrhea and quality of life of patients in comparison to the control group, which was administered a placebo [27]. Also, the authors of the study themselves pointed out the possibility of a bias being present due to a low number of test subjects.

As another method of managing sialorrhea, the radiotherapy targeted on head and neck was proposed, namely due to its secondary effect discovered during treating cancer patients [28]. When specifically targeted on the salivary glands of patients with ALS, gland cells damaged by the ionising radiation ceased excretion of saliva with a great positive effect on the patient’s quality of life, based upon a good response to the treatment in 16 out of 18 patients.

The most invasive method of sialorrhea management would be a surgical removal of salivary glands or adjacent salivary ducts [29], a method no longer widely in use due to the overall low life expectancy of ALS patients. Therefore, surgical solution of sialorrhea is only considered if no other treatment of sialorrhea has been found effective. In practice, in order to prevent dry mouth syndrome, only one of the pair of salivary glands is usually operated upon, as the remaining gland produces enough saliva.

### 1.7. Drug-Based Management of ALS

Riluzole, a drug approved as a general treatment of ALS, was found to extend the mean survival rate of patients by 2 to 3 months if a daily dose of 100 mg is administered [30]. Furthermore, if the treatment via riluzole was commenced in earlier stages of ALS, the onset of symptoms was postponed. Mechanism-wise, riluzole lowers the release of glutamatergic neurotransmitter by inhibiting the release of presynaptic voltage-gated Na^+^ channels, with presynaptic calcium channel inhibition also implied [31].

In contrast to the improved life-expectancy of patients taking riluzole, the neuroprotective attenuation of synaptic function causes several adverse side effects, such as weakness, dizziness, nausea, diarrhoea and cough [32]. If the medication is stopped, side effects disappear soon after, but together with the therapeutic effect of riluzole as well. Furthermore, riluzole in combination with birth control, drugs for treating infections, high cholesterol, seizures, pain or arthritis was found to cause liver damage [33]. The marginal effectiveness of riluzole, together with its series of adverse side effects, has led to a reduction in its use.

Recently, replacement drugs to riluzole are being considered, for example, Tofersen, an antisense oligonucleotide designed to inhibit expression of *SOD1*, in effect, potentially treating cases of ALS caused by said gene [34], although the effectiveness of Tofersen is still a matter of research [35].

Susceptibility of patients to bacterial infections of the respiratory apparatus [36] is another health complication typically accompanying ALS, while not being directly caused by it. The risk is elevated in patients suffering from ALS due to the already mentioned increased production of mucus and saliva. Sialorrhea in combination with food particles entering the lower airways due to the atrophy of epiglottic muscles provides an ideal environment for colonisation of the patient’s respiratory apparatus by pathogenic bacteria, further restricting the already limited respiration and subsequently decreasing the patient’s quality of life.

Although the use of antibiotics might further negatively impact the composition of the patient’s intestinal microbiome and therefore accelerate the progression of the disease, antibiotics are vital for the patient’s survival at these late stages of ALS.

### 1.8. Non-Invasive Ventilation Support

Chronic neuromuscular respiratory failure is the most common cause of mortality and morbidity in ALS. The main cause of the respiratory failure is progressive weakness of the diaphragm [37]. Additionally, expiratory muscles are also affected, which is detrimental to the maintenance of airway clearance and management of secretion, complications that further negatively reflect on the patient’s prognosis. Some improvement was observed in patients who underwent respiratory strength training [38], but other means of ventilation support are often later recommended as the disease progresses.

In order for a patient to be put on non-invasive ventilation support, at least one criterion from the list must be met [37]:

- Forced vital capacity is less than 50%.
- Maximal inspiratory pressure is less than −60 mm Hg.
- During overnight oximetry, the oxygen saturation was measured at less than 88% for noncontinuous 5 min periods.
- Arterial blood gas PCO_2_ was more than 45 mm Hg.

The non-invasive ventilation support extended the life expectancy of patients on average by 205 days over those whose respiration was not supported by external means [39]. In order to achieve a greater effect, an invasive option must be considered, for example, tracheostomy, which extends the life expectancy of patients by 1 to 2 years in comparison to patients without any respiratory support [40].

### 1.9. Intestinal Microbiome as a Path to Treatment of ALS

Based upon the relation of the intestinal microbiome and the pathogenesis of ALS, it was proposed that an intentional intervention aimed at its composition might have a positive effect on the course of ALS and also on other neurodegenerative disorders, for example, Parkinson’s disease, Alzheimer’s disease, Huntington’s syndrome, and neuropathological autism [41].

An example of said intervention into the human intestinal microbiome for the purpose of altering its composition as a means of therapy would be faecal microbiota transplantation, a procedure during which faecal matter sampled from a donor’s gastrointestinal tract is transplanted into that of a recipient in order to achieve a positive effect on the recipient’s health [42]. FMT can be administered in the form of perioral capsules, which allow for a non-invasive application of microbiota, a better control over the dosage, and, comfort-wise, are well received by patients [43].

WMT is used as an effective means of intestinal microbiome reconstruction and might show itself as a promising therapeutic approach in the treatment of selected diseases [44].

Contemporarily, FMT and its variants are routinely used as a treatment of recurrent infections by *Clostridium difficile* (CDI) following an exhaustion of the patient’s intestinal microbiome via antibiotics in order to allow a recolonisation of the intestines with a healthy microbiome unaffected by the infection [45].

In neurodegenerative disorders, it is expected of FMT to restore biodiversity of the patient’s microbiome and therefore effectively remove its dysbiosis as a factor involved in their pathogenesis, either betterment of symptoms or outright curing these diseases in some cases.

In the past, the effects of FMT and its variants on the prognosis of ALS were studied only on animals, namely on mouse models of ALS, but after the conclusion of the necessary preclinical studies, researchers expanded the experimentation onto human patients.

### 1.10. Preclinical Studies on Animals

In the last decade, several groups have shown that gut microbiota and gut barrier dysfunction can modify disease course in ALS mouse models, providing the main rationale for FMT as a therapeutic strategy [46]. Importantly, only a small number of published preclinical ALS studies actually use full FMT. Most others manipulate the microbiome with antibiotics, defined bacterial strains, or metabolites rather than whole-stool transfer.

Wu et al. [47] used transgenic SOD1-G93A mice (classic familial ALS model) and assessed gut morphology, tight junction proteins, and faecal microbiota before and after symptom onset. Even before motor symptoms, ALS mice showed shortened villi and disrupted tight junction proteins, increased intestinal permeability (“leaky gut”), and significant dysbiosis, including loss of butyrate-producing bacteria. These changes worsened as the disease progressed, in parallel with neuroinflammation and motor decline.

Wu et al. [47] established that gut barrier breakdown and early microbiota changes are part of ALS pathophysiology, not just a late consequence of paralysis. This set the stage for later interventional studies (including FMT) aimed at restoring microbiome and barrier function.

Zhang et al. [48] also used the SOD1-G93A mice. The mice received 2% sodium butyrate in drinking water before symptom onset and continuing through their lives. This study improved intestinal barrier integrity and Paneth cell morphology, reduced misfolded SOD1 aggregation in the spinal cord, and also delayed disease onset and extended survival compared with untreated ALS mice. This work showed that targeting gut dysbiosis can directly modify ALS disease course in mice, strengthening the biological plausibility that a more global microbiota reset via FMT could be beneficial.

Blacher et al. [49] used *SOD1* mice plus faecal and plasma samples from ALS patients and combined metagenomics and metabolomics to link particular bacteria and metabolites (notably nicotinamide) to disease severity. Their study provided mechanistic evidence that specific commensal species and their metabolites can be protective in ALS. Although they did not do a full FMT, the work supports the idea that transplanting a “healthier” microbiome (via FMT) might supply missing beneficial taxa and metabolites.

Burberry et al. [50] used mice deficient in *C9orf72*, the gene commonly mutated in familial ALS and frontotemporal dementia (FTD). Burberry et al. [50] showed causally that gut microbiota composition can tune inflammation and lifespan in an ALS-relevant genetic model, and that FMT from a “good” microbiome can rescue a “bad” one. This is a central preclinical proof-of-concept for ALS-directed FMT.

### 1.11. Outcomes from FMT in Human Patients

Based on promising results shown by studies investigating the effect of FMT on ALS conducted on mouse models, in recent years, several research teams proceeded to clinical studies on human patients.

A search was conducted in PubMed, WOS, and ClinicalTrials.gov on the topic amyotrophic lateral sclerosis and faecal microbiota transplantation/faecal microbiota transplantation and Washed microbiota transplantation, excluding experimental studies and studies related to other neurodegenerative diseases (e.g., Parkinson’s disease, Alzheimer’s disease, Multiple Sclerosis, and Motor neuron disease), as well as other diseases (e.g., autism, gastrointestinal and extraintestinal disorders, ischemic stroke, skin cancer).

To date, three of these studies already published their findings and two studies are being conducted: NCT03766321 “FETR-ALS Study Protocol: A Randomized Clinical Trial of Fecal Microbiota Transplantation in Amyotrophic Lateral Sclerosis” (see Mandrioli et al., [51], with 28 patients at the Azienda Ospedaliero-Universitaria di Modena, Italy (completed and finishing the data), and NCT07017946 “Intestinal Microbiome Transplant in ALS” Duke University, North Carolina, US (estimated 20 patients, not yet recruiting). For an overview, see Table 1.

#### 1.11.1. Washed Microbiota Transplantation Stopped the Deterioration of Amyotrophic Lateral Sclerosis: The First Case Report and Narrative Review [52]

The earliest study examining the effects of altering the intestinal microbiome in ALS patients was conducted in Nanjing, China [52]. The subject was a 48-year-old woman who initially exhibited muscle stiffness and weakness, accompanied by moderate constipation, followed by the manifestation of amyotrophy. After an extensive examination ruled out other possible diseases, the patient was diagnosed with ALS. Despite standard treatment with riluzole, baclofen, coenzyme Q10, and vitamin E being attempted, symptoms rapidly progressed.

In response to disease progression, washed microbiota transplantation (WMT), a variant of FMT, was proposed as a treatment for constipation, with the potential beneficial effects on ALS also considered. Unlike FMT, during WMT, larger faecal particles, fibre, and undigested food are filtered out during preparation of the transplant to reduce the unpredictability in microbiome studies due to inherent heterogeneity [55,56]. Additionally, WMT is expected to minimise the risk of negative side effects occurring compared to FMT.

In the Nanjing study, WMT was administered via mid-gut or colonic transendoscopic enteral tube (TET), a procedure where a flexible tube is colonoscopically inserted and left in place for the duration of the treatment. This allows for repeated WMT or FMT administration without repeated colonoscopies [57].

As a result of therapy, constipation was brought under control, and improvements in both ALSFRS-R and ALSAQ-40 scores were observed [52]. Also, the progression of ALS symptoms plateaued, and the patient gradually regained her balance and gait, which had been impaired by reduced muscle tone. Several months after WMT administration, the patient suffered a head injury, leading to a rapid worsening of her symptoms and resulting in wheelchair dependence. Repeated WMT therapy once again halted ALS progression, followed by health improvements, including regaining the ability to stand with assistance and improved muscle tone.

#### 1.11.2. Faecal Microbiota Transplantation Significantly Improved Respiratory Failure of Amyotrophic Lateral Sclerosis [53]

The second study originated in Peking, China [53], involving two male ALS patients aged 71 and 76. FMT was administered after conventional treatments had little or no effect. The faecal donor was a 28-year-old woman, and samples were cryogenically frozen for long-term storage. FMT was delivered after a routine colonoscopy via endoscope into the terminal ileum. Both patients underwent two treatment sessions about four weeks apart, with effect assessed using the ALSFRS-R scale.

Within five days of the first FMT session, the first patient (71) was able to be switched from mechanical ventilation to non-invasive support, and the second patient (76) also showed improved respiration. Both patients exhibited increased muscle strength, with the first patient regaining the ability to stand with assistance.

On a molecular level, subsequent metagenomic and metabolic analysis revealed increased levels of metabolites involved in arginine biosynthesis and reduced levels of metabolites of branched-chain amino acid (BCAA) biosynthesis. Additionally, the intestinal microbiome composition of both patients shifted toward that of the donor.

#### 1.11.3. Effect of Faecal Microbiota Transplantation on Patients with Sporadic Amyotrophic Lateral Sclerosis: A Randomised, Double-Blind, Placebo-Controlled Trial [54]

The third and largest study was conducted in Zhengzhou, China [54]. This randomised study included 27 participants of both sexes, represented proportionally by men and women, where 14 received FMT, and 13 received a placebo. The progression of ALS in the reaction to FMT was primarily assessed using the ALSFRS-R scale, with secondary outcomes including gastrointestinal function, muscle strength, autonomic nervous system function, cognition, quality of life, gut microbiome composition, and plasma neurofilament light chain protein. Prior to FMT, patients’ native gut microbiomes were depleted with seven days of ciprofloxacin and metronidazole, followed by transplantation via TET into the ileocecum after screening colonoscopy. The study found no significant slowing of disease progression in FMT-treated patients compared to the control group.

## 2. Discussion

This work provides a focused comparative synthesis of the currently available human clinical evidence on FMT in ASL patients, an area where only three human studies have been published to date, alongside two ongoing trials: one in Italy and one in the USA. Conflicting results have been evidenced. While increased quality of life was observed in three patients in Nanjing [52] and Peking [53], the largest study from Zhengzhou, which consisted of 27 subjects, reported no significant improvement in patients’ health. The main methodological difference between the smaller studies and the larger one from Zhengzhou [54] was the deliberate microbiome depletion prior to FMT administration, which may have caused reduced treatment efficacy compared to two previous studies.

In the Nanjing case [52], health improvements after treatment were observed twice, the second following a severe deterioration of ALS symptoms after head injury. Since this was a single case, it cannot be ruled out that the second improvement was due to post-injury recovery rather than ALS treatment.

Another point to be considered is the effect of cryogenic storage on the viability of microbiome samples. While human gut microbiotas are adapted to body temperature, contemporary studies found no significant negative effects of long-term storage at up to −80 °C on microbial biodiversity [58].

The administration method also deserves attention, whether colonoscopy could be replaced by less invasive procedures. In treating recurrent *Clostridium difficile* infections, the efficacy of colonoscopic FMT has been compared with oral capsules containing the faecal transplant, and both methods showed similar effectiveness in restoring the gut microbiome [59]. Unlike capsules, colonoscopy allows for targeted delivery to sites such as the cecum, the region with the highest colonic microbial diversity [60]. A compromise is TET [61], which requires only one colonoscopy to position a semi-permanent tube into the targeted area of intestines for repeated FMT and its variants administration, improving patient comfort over repeated colonoscopies.

Future research should also focus on the effects of FMT on different ALS phenotypes. As disease progression is heavily influenced by the underlying genetic mutation involved in this pathogenesis, FMT may not be effective in all ALS cases. This research could be facilitated by whole-genome sequencing, now feasible on a large scale, thanks to recent advances in DNA sequencing technologies. Likewise, future studies should compare long-term microbiome changes between healthy individuals and those with ALS.

It is also important to replicate FMT studies in non-Asian populations, owing to the high heterogeneity of the human gut microbiome, shaped by environment, diet, and genetics. Even minor differences in these factors can significantly influence the effectiveness of microbiome-targeted therapies across populations.

## 3. Conclusions

To date, the largest published study has not found a significant effect of FMT on ALS progression, but individual cases suggest that FMT is a promising avenue for ALS treatment. Future research should replicate existing studies, focusing on FMT’s effects on ALS variants influenced by patient genotype, and expand to non-Asian populations due to microbiome heterogeneity. Even if FMT does not prove to be a causal treatment for ALS, it may still provide relief of accompanying symptoms and associated health issues.

## Figures and Tables

**Table 1:** Selected demographic information from studies focusing on the effect of FMT in ALS.

Continent/Country	Nr. of Patients	Treatment/ Placebo	Sex	Age	Intervention/Treatment (Administration)	Study Duration	Status
Asia/China	1	1/0	♀	<50	WMT (TET)	12 m. follow-up	Published [52]
Asia/China	2	2/0	♂	>70	FMT (colonoscopy)	10 & 17 weeks	Published [53]
Asia/China	27	14/13	all	18–65	FMT (TET)	6 months	Published [54]
Europe/Italy	42	28/14	all	18–70	FMT/Placebo (nasojejunal tube)	6 months	NCT03766321—closed— unpublished outcomes (Azienda Ospedaliero-Universitaria di Modena, 2024)
North America/ USA	20	20/0	all	18+	MTP-101C (p.o. drug)	24 weeks	NCT07017946 -planned (Duke University)

## Data Availability

No new data were created in this study.
